# Elevated colorectal cancer incidence among American Indian/*Alaska* native persons in *Alaska* compared to other populations worldwide

**DOI:** 10.1080/22423982.2023.2184749

**Published:** 2023-03-03

**Authors:** Donald Haverkamp, Diana Redwood, Elena Roik, Stephen Vindigni, Timothy Thomas

**Affiliations:** a Epidemiologist, Centers for Disease Control and Prevention Albuquerque, NM, USA; b Program Manager, Alaska Native Tribal Health Consortium, Anchorage, AK, USA; c Program Director, Alaska Native Tribal Health Consortium, Anchorage, AK, USA; d Gastroenterologist, Alaska Native Tribal Health Consortium, Anchorage, AK, USA; e Department of Gastroenterology, University of Washington, Seattle, WA, USA; f Research Services Director, Alaska Native Tribal Health Consortium, Anchorage, AK, USA

**Keywords:** Native american, cancer surveillance, colorectal cancer, Alaska native, colon cancer, health disparities, American Indian

## Abstract

Colorectal cancer (CRC) is a leading cancer worldwide; incidence varies greatly by country and racial group. We compared 2018 American Indian/Alaska Native (AI/AN) CRC incidence rates in Alaska to other Tribal, racial, and international population rates. AI/AN persons in Alaska had the highest CRC incidence rate among US Tribal and racial groups (61.9/100,000 in 2018). AI/AN persons in Alaska also had higher rates than those reported for any other country in the world in 2018 except for Hungary, where males had a higher CRC incidence rate than AI/AN males in Alaska (70.6/100,000 and 63.6/100,000 respectively). This review of CRC incidence rates from populations in the United States and worldwide showed that AI/AN persons in Alaska had the highest documented incidence rate of CRC in the world in 2018. It is important to inform health systems serving AI/AN persons in Alaska about policies and interventions that can support CRC screening to reduce the burden of this disease.

Previous reports have noted markedly higher colorectal cancer (CRC) incidence rates among American Indian and Alaska Native (AI/AN) persons residing in Alaska, compared to AI/AN populations residing in all other regions of the country, and compared to non-Native populations in the United States (US) [1–3]. CRC was the most commonly diagnosed cancer among Alaska Native men and women from 2014–2018 [3]. There has also been a steep increase in early onset colorectal cancer among AI/AN in Alaska ages 20–49, which increased 5.2% annually between 1996 and 2019 [4].

In 2020, there were an estimated 148,085 AI/AN persons living in Alaska, which was 19% of the total Alaskan population [5]. In this brief report, we present recent CRC incidence rates among AI/AN persons in Alaska, and compare them to rates among other racial groups in Alaska and AI/AN persons in other regions of the US, and to published CRC incidence rate estimates for other countries around the world.

## Methods

To calculate CRC incidence in the US, we used U.S. Cancer Statistics [6] data, which includes cancer registry data from the Centers for Disease Control and Prevention’s (CDC) National Program of Cancer Registries (NPCR) [7] and the National Cancer Institute’s (NCI) Surveillance, Epidemiology, and End Results (SEER) [8] Program. Cancer data for AI/AN persons in Alaska came from the Alaska Cancer Registry as well as the Alaska Native Tumor Registry, which is a member of the SEER program. This population-based central cancer registry records information on AI/AN persons who meet eligibility requirements for Indian Health Service benefits [9], who have been diagnosed with cancer in Alaska since 1969, and who resided in Alaska at the time of diagnosis.

**Figure 1.**
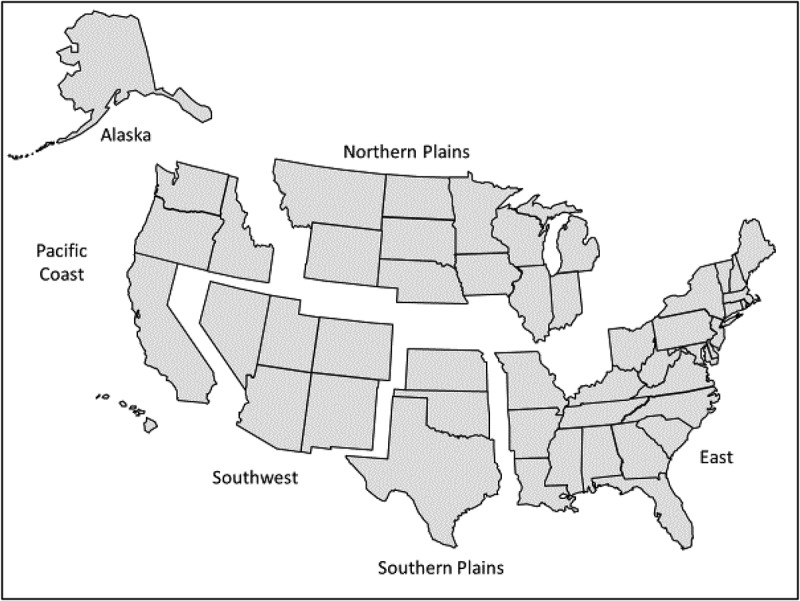
Map of United StatesIndian health service regions.

CRC rates among AI/AN populations were calculated for six Indian Health Service regions in the US (Figure 1), and expressed per 100,000 population, using SEER*Stat software [10]. To examine rates by race in Alaska (Table 2), 5 years of data were aggregated because of the low number of cases in any single year for some racial groups. Rates were age-adjusted using the World Health Organization World Standard Population (2000–2025) [11], so that rates for AI/AN persons in the US could be compared with rates that have been estimated for countries around the world. Joinpoint [12] regression analysis was used to identify trends and quantify annual percent change in the CRC incidence rates among AI/AN and White persons in Alaska.

Estimates of worldwide CRC incidence were from the International Agency for Research on Cancer Global Cancer Observatory GLOBOCAN 2018 database. GLOBOCAN provides estimates of incidence rates, mortality rates, and cancer prevalence in 185 countries or territories for 36 cancer types by sex and age group [13].

Institutional review board approval and informed consent were not required for the current study because all NPCR, SEER and GLOBOCAN data are publicly available and collected for surveillance purposes, and all data were de-identified. Tribal review and approval were obtained for publication of this report from the Alaska Native Tribal Health Consortium, which is the statewide Tribal health organization serving all 229 federally recognised Tribes and all Alaska Native and American Indian individuals in Alaska.

## Results

There was wide regional variation in CRC incidence rates among AI/AN populations within the US, with AI/AN persons in the Alaska region having a higher rate than all other US Indian Health Service regions (Table 1). The CRC incidence rate among AI/AN persons in the Alaska region in 2018 was 61.9 per 100,000 persons (63.6/100,000 among males, 59.8/100,000 among females) (Table 1). This rate was nearly twice the CRC incidence of the next highest group, the Southern Plains region (31.1/100,000), and over five times as high as the lowest group, the East region (11.3/100,000).

**Table 1.** Age-adjusted colorectal cancer incidence rates,^a^ counts, and population for American Indian/Alaska Native persons, by United States region, all counties, 2018.

Region		Rate	SE	Lower CI	Upper CI	Count	Population
Alaska	Male and female	61.9	7.0	49.0	77.2	81	128,287
Alaska	Male	63.6	10.2	45.2	87.0	40	64,917
Alaska	Female	59.8	9.5	42.6	81.6	41	63,370
Southern Plains	Male and female	31.1	1.9	27.4	35.1	265	789,140
Southern Plains	Male	36.0	3.0	30.3	42.4	145	396,146
Southern Plains	Female	26.9	2.5	22.3	32.3	120	392,994
Northern Plains	Male and female	22.9	1.9	19.4	27.0	148	654,135
Northern Plains	Male	27.6	3.1	21.9	34.3	84	326,219
Northern Plains	Female	19.0	2.4	14.6	24.3	64	327,916
Southwest	Male and female	18.2	1.5	15.4	21.3	152	813,988
Southwest	Male	19.1	2.3	14.9	24.0	72	401,544
Southwest	Female	17.4	2.0	13.8	21.7	80	412,444
Pacific Coast	Male and female	15.0	1.2	12.8	17.5	172	1,082,582
Pacific Coast	Male	15.9	1.7	12.7	19.7	85	546,143
Pacific Coast	Female	14.4	1.6	11.6	17.9	87	536,439
East	Male and female	11.3	0.9	9.7	13.2	166	1,208,075
East	Male	11.8	1.3	9.4	14.7	84	609,562
East	Female	10.9	1.2	8.7	13.6	82	598,513
All regions	Male and female	19.3	0.6	18.1	20.5	984	4,676,207
All regions	Male	21.2	0.9	19.4	23.2	510	2,344,531
All regions	Female	17.7	0.8	16.1	19.4	474	2,331,676

^a^All data age-adjusted using the World Health Organization’s World Standard Population (2000–2025).

The incidence rate among AI/AN persons in Alaska also exceeded rates for every other racial group in Alaska (Table 2). During 2014–2018, the AI/AN rate in Alaska (67.1/100,000) was over twice as high as the rate for the White population in Alaska (26.4/100,000), and over three times higher than the rate among Asian/Pacific Islander persons and Black persons in Alaska (20.3/100,000 and 17.1/100,000, respectively). Among every racial group in Alaska, males had higher CRC incidence rates than females.

**Table 2.** Age-adjusted colorectal cancer incidence rates, counts, and population for American Indian/Alaska Native persons by race, Alaska, 2014–2018.

Sex	Race^a^	Rate^b^	Lower CI	Upper CI	Count	Population
Male and female	All races	31.3	29.6	32.9	1,462	3,690,076
Male and female	White	26.4	24.7	28.2	942	2,567,692
Male and female	Black	17.1	10.7	25.7	24	178,375
Male and female	AI/AN	67.1	60.8	73.9	420	636,660
Male and female	API	20.3	15.8	25.7	71	307,349
Male	All races	33.5	31.2	36	803	1,929,578
Male	White	29.5	27	32.1	552	1,359,708
Male	Black	19.9	10.2	34.5	14	98,791
Male	AI/AN	67.2	58.1	77.3	200	322,706
Male	API	23.0	15.8	32.5	34	148,373
Female	All races	28.9	26.7	31.2	659	1,760,498
Female	White	23.1	20.8	25.6	390	1,207,984
Female	Black	14.4	6.8	26.5	10	79,584
Female	AI/AN	66.7	58.1	76.2	220	313,954
Female	API	19.0	13.3	26.3	37	158,976

^a^All rates per 100,000 and age-adjusted using the World Health Organization’s World Standard Population (2000–2025).

^b^Non-Hispanic and Hispanic included.

AI/AN = American Indian/Alaska Native; API = Asian or Pacific Islander

**Figure 2.**
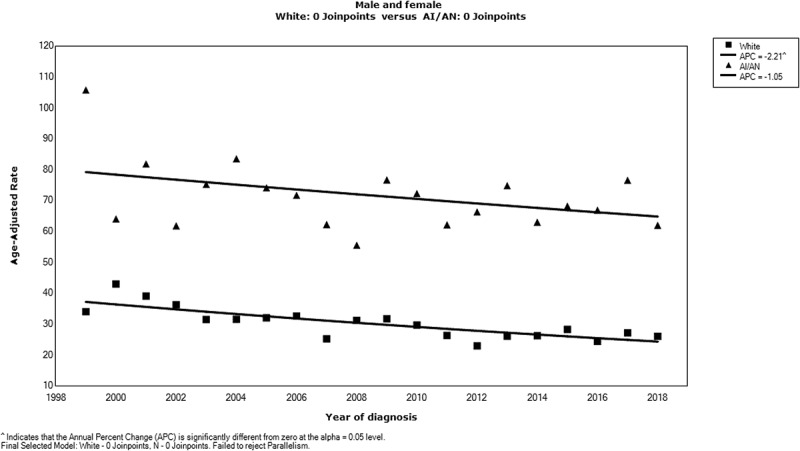
Trends in colorectal cancer incidence rates, American Indian/Alaska Native (AI/AN) persons and White persons, Alaska, 1999–2018.

From 1999 to 2018, the CRC incidence rate among AI/AN persons in Alaska did not decline significantly, whereas the White population in Alaska experienced a significant decrease in the CRC incidence rate during the same period (Figure 2).

Compared internationally, the CRC incidence rate among AI/AN persons in Alaska (61.9/100,000 in 2018) was higher than the rate for any country with known CRC incidence rates that year and 1.2 times higher than the highest ranked country, Hungary (51.2/100,000)(Table 3). The rate among AI/AN females in Alaska (59.8/100,000) was 1.5 times higher than females in Norway, the country with the highest rate for females (39.3/100,000) (Table 3). Among males, the CRC incidence rate in Hungary was the only rate higher than AI/AN males in Alaska (70.6/100,000 and 63.6/100,000 respectively)(Table 3).

**Table 3.** Top 10 colorectal cancer incidence rates worldwide, by men and women, 2018*.

Rank	Country	Age-standardised rate per 100,000
Men and Women, combined		
1	Hungary	51.2
2	South Korea	44.5
3	Slovakia	43.8
4	Norway	42.9
5	Slovenia	41.1
6	Denmark	41.0
6	Portugal	40.0
8 =	Barbados	38.9
8 =	Japan	38.9
10	Netherlands	37.8
Women		
1	Norway	39.3
2	Hungary	36.8
3	Denmark	36.6
4	Singapore	34.0
5	Australia	32.4
6	South Korea	31.3
7	Slovakia	31.2
8	Netherlands	31.1
9	New Zealand	30.8
10	Japan	29.6
Men		
1	Hungary	70.6
2	Slovakia	60.7
3	South Korea	59.5
4	Slovenia	58.9
5	Portugal	54.0
6	Barbados	50.3
7	Japan	49.1
8	Serbia	49.0
9	Moldova	47.3
10	Norway	46.9

*Data source: World Health Organization/International Agency for Research on Cancer, Global Cancer Observatory, GLOBOCAN 2018 database (https://gco.iarc.fr).

## Discussion

This review of Tribal and non-Tribal populations in the US showed that AI/AN persons in Alaska had the highest documented CRC incidence rate compared with rates for AI/AN persons in any other region in the country in 2018, and the highest rate compared with all other racial groups in Alaska during 2014–2018. The high rate among AI/AN persons in Alaska has persisted over time, despite declining CRC incidence rates among Alaska non-Native populations and the overall US population [14]. In the US, there was a 3.3% annual decrease in CRC incidence in the late 2000s coinciding with increasing CRC screening with colonoscopy among adults aged 50 or older [1]. Our analyses and others [15] show that there has been no significant decrease in the CRC incidence rate among AI/AN in Alaska during the past 2 decades. Our comparison of CRC incidence rates among AI/AN persons in Alaska, compared with published country-level CRC incidence rates worldwide [16,17], showed that AI/AN persons in Alaska (males and females combined) had the highest documented CRC incidence rate in the world in 2018.

There have been notable declines in CRC incidence and mortality in countries with decreases in smoking and increased uptake of screening by colonoscopy with polypectomy [18–20]. Through screening, CRC can be detected early, when it is more treatable, and can be prevented if precancerous polyps are found and removed during screening [21]. Because of this, there has been an effort to increase screening for CRC among AI/AN persons in Alaska and screening is a covered service within the Alaska Tribal Health System [22,23]. According to Alaska Behavioral Risk Factor Surveillance System data, the percentage of Alaska Native persons in Alaska who were up-to-date with CRC screening in 2020 was 71.1% (95% CI: 62.4, 78.5) [24].

CRC incidence rates tend to rise in countries experiencing rapid socio-economic development and shifts in dietary behaviours and living conditions [25–27]. AI/AN persons in Alaska have experienced a shift from traditional diet and physical activity patterns to more reliance on store-bought foods, and they have a higher incidence of chronic diseases, including heart disease and cancer [28–30]. This health transition is similar to that experienced by other Arctic Indigenous populations [31,32].

Studies have shown CRC incidence rates to be higher among Indigenous persons than non-Indigenous populations in some places, such as Ontario, Canada [33], although lower than non-Indigenous populations in other locations, such as China [34] and Scandinavia [32,35]. However, many countries do not separate out CRC incidence among Indigenous groups in their national statistics; thus, it was not possible in this study to compare rates among AI/AN populations in Alaska to other Indigenous populations worldwide. This is an area of inquiry that would be helpful to explore, to discover if there are other Tribal populations that also experience higher CRC incidence rates than non-Tribal groups within their home country.

High-quality cancer registry data are not available in most low- and middle-income countries, which is another limitation of our comparison to CRC incidence rates in other countries [16]. Therefore, there may be populations that have higher rates and estimates than those included in this study. Racial misclassification can also contribute to inaccuracies in reported cancer incidence and mortality [36]. The validity and quality of national estimates depends upon the representativeness of the source information, which is important for planning and implementing evidence-based cancer control programs [13,16].

In addition, the rates among AI/AN persons in Alaska presented here are not comparable with previous publications in which rates were calculated using the 2000 US Standard Population [2,37]. For the purpose of being able to compare rates of other countries, we used the World Health Organization World Standard Population (2000–2025) [11] to calculate incidence rates. The age distribution of the World Standard Population reflects a much younger population than the 2000 US Standard Population [38], and this may have lessened the magnitude of the observed differences in rates between AI/AN persons in Alaska and other countries.

Reasons for the extremely high CRC incidence rate among AI/AN persons in Alaska are not well understood and may be related to behavioral factors known in other populations to be associated with CRC risk [20], including tobacco use, physical inactivity, low fruit and vegetable intake, and alcohol consumption [39]. There may be additional environmental factors associated with this higher risk, such as infection with *Helicobacter pylori*, which has been associated with development of CRC [40,41].

To help reduce the burden of this preventable and treatable disease, it is important to continue to inform AI/AN communities about the social and environmental factors that may limit healthy behaviours [42], and to guide health systems serving AI/AN persons about policies and interventions that can support CRC screening.

## Abbreviations

CRC = colorectal cancer

AI/AN = American Indian/Alaska Native

CDC = Centers for Disease Control and Prevention

NCI = National Cancer Institute

NPCR = National Program of Cancer Registries

SEER = Surveillance, Epidemiology, and End Results
